# Gaze Focalization System for Driving Applications Using OpenFace 2.0 Toolkit with NARMAX Algorithm in Accidental Scenarios

**DOI:** 10.3390/s21186262

**Published:** 2021-09-18

**Authors:** Javier Araluce, Luis M. Bergasa, Manuel Ocaña, Elena López-Guillén, Pedro A. Revenga, J. Felipe Arango, Oscar Pérez

**Affiliations:** Electronics Department, University of Alcalá, 28801 Alcalá de Henares, Spain; luism.bergasa@uah.es (L.M.B.); mocana@depeca.uah.es (M.O.); elena.lopezg@uah.es (E.L.-G.); pedro.revenga@uah.es (P.A.R.); juanfelipe.arango@uah.es (J.F.A.); oscarcanre@gmail.com (O.P.)

**Keywords:** driver focalization, accidental scenarios, gaze estimation, heat map, computer vision, NARMAX

## Abstract

Monitoring driver attention using the gaze estimation is a typical approach used on road scenes. This indicator is of great importance for safe driving, specially on Level 3 and Level 4 automation systems, where the take over request control strategy could be based on the driver’s gaze estimation. Nowadays, gaze estimation techniques used in the state-of-the-art are intrusive and costly, and these two aspects are limiting the usage of these techniques on real vehicles. To test this kind of application, there are some databases focused on critical situations in simulation, but they do not show real accidents because of the complexity and the danger to record them. Within this context, this paper presents a low-cost and non-intrusive camera-based gaze mapping system integrating the open-source state-of-the-art OpenFace 2.0 Toolkit to visualize the driver focalization on a database composed of recorded real traffic scenes through a heat map using NARMAX (Nonlinear AutoRegressive Moving Average model with eXogenous inputs) to establish the correspondence between the OpenFace 2.0 parameters and the screen region the user is looking at. This proposal is an improvement of our previous work, which was based on a linear approximation using a projection matrix. The proposal has been validated using the recent and challenging public database DADA2000, which has 2000 video sequences with annotated driving scenarios based on real accidents. We compare our proposal with our previous one and with an expensive desktop-mounted eye-tracker, obtaining on par results. We proved that this method can be used to record driver attention databases.

## 1. Introduction

In recent years, there have been important advances in the intelligent vehicles field with the implementation of the autonomous driving as one of the most challenging and difficult tasks on this research field, from an academic and industrial point of view. This automation can be divided into five Levels according to SAE (J3016) [4], achieving full autonomy at Level 5. On the latest vehicle models, we have different Advanced Driver Assistance Systems (ADAS) that are in charge of different driving tasks that so far have been done by the driver. In the first two levels of automation, the driver must be fully engaged during the driving, supervising the vehicle and the actions done by it, under all circumstances. In the next two levels (3 and 4), the driver may be not on the driving loop in some situations or during the entire path if the vehicle is able to drive in all the circumstances that could happen on the route. Level 5 does not present any problem because the vehicle is able to drive under all circumstances without human intervention. It is on the above levels where the problems arise, because the driver must be on the loop, but is not aware of the entire surrounding area (Level 1 and 2) or is out of the loop and needs to take over control for some unexpected reasons (Level 3 and 4), bringing back them into the loop [5].

In this future driving world, where shared control between human and machine will be present, ascertaining that the state of driver is suitable for driving will be a key task for safety driving reasons. Specially, when the vehicle requests driver assistance in a complex scenario where it is not able to handle it because of any issue. In these scenarios where the vehicle requests human help, it is crucial to ascertain that the driver is aware of their surroundings (e.g., surrounding pedestrians or vehicles) just before the take over request to perform a safety transition. For this reason, the driver’s visual focalization must be evaluated to ensure that awareness of their surroundings, and it must be a key task in the development of automated vehicles. For this evaluation, gaze tracking estimation is the most used approach to evaluate driver’s visual focalization [6].

There are different approaches in the literature about how gaze tracking should be done, mainly based on head-mounted eye trackers [7,8,9], active desktop-mounted eye trackers [10] and passive desktop-mounted cameras [6,11,12,13,14], which track the gaze. The first ones provide accurate gaze information, but they are intrusive and costly. The latter are low-cost and non-intrusive, but less accurate.

In work [15], the authors made a review of the different methods of eye tracking and gaze tracking, which are two different areas of research. However, as the main contribution of this paper is to present gaze tracking, we will focus on this part. Gaze tracking systems based on cameras use two different approaches: model-based techniques and regression-based methods. The former ones use a geometric model of the eye to calculate the gaze direction. Gaze focalization is obtained by the intersection of the gaze vector with the object [16,17,18,19,20]. The later ones [21,22,23,24,25] map the image features to the gaze coordinates using neural networks, as in [26], or polynomials, as in [27]. Most of these works use IR lights to obtain a stable gaze, whose usage for a long period of time causes dryness and fatigue in the eyes [28].

We propose the use of a regression-based system, but using the parameters extracted from a face tracker OpenFace 2.0 [1] to obtain the gaze focalization over a screen as our visual attention system.

OpenFace 2.0 is a facial behavior analysis toolkit. In [1], authors made a wide comparison with other state-of-the-art systems. As our proposal is based on gaze focalization estimation, we will focus on this topic. Some state-of-the-art gaze estimators are: WebGazer.js [29], EyeTab [30] and OKAO [31]. This last tool is not free to use, which does not follow our policy of Open Source. Regarding the former two systems, they do not include head position, which is used in our algorithm to estimate the gaze. Moreover, OpenFace 2.0 provides a C++ code and a simplified model that allows real-time operation, which is a key factor in our proposal. This paragraph explained the comparison of OpenFace 2.0 with other systems has been added in the text (line xx to line yy).

Usually, visual attention systems are validated in simulation with simple scenes. However, the simulation of driver attention in complex driving scenarios is rather challenging and highly subjective [3]. These systems aim to automatically estimate the area where the driver is focusing their attention. For this reason, the acquisition systems and computer vision techniques are one step ahead from traditional human factor approaches focused on driving behavior understanding using manual tools. In this research field, there are different databases that collect driver’s attention in the state-of-the-art. The first public database based on driving attention maps was the project DR(eye)VE [32], which collected 555,000 frames with those attention maps generated by the actual driver. After that, Berkeley DeepDrive Attention (BDDA) database [10] was published, which is formed by 1232 videos that produced a braking event and the attention map was recorded later in-lab. This approach not only presented a critical situation where the attention is important, but also the attention map generated by more than one user for the scene. However, these scenes are not challenging enough to test the attention because these situations do not cause accidents. Therefore, we have evaluated our system on the recent DADA2000 database [3], which is the largest and most diverse driving attention database composed by accidental scenarios. It contains 2000 video clips annotated with the accident localization and the attention map generated in-lab by some users with fairly complex accidental scenarios in diverse weather and lighting conditions.

Previously, a slight calibration method based on a linear calibration using a simple projection matrix was proposed by the authors in [2]. It was formed by a low-cost and non-intrusive camera-based gaze mapping system able to estimate the driver focalization on challenging prerecorded traffic scenes through a heat map. Gaze direction was calculated by using the open-source state-of-the-art OpenFace 2.0 Toolkit [1] combined with a projection matrix.

This paper presents an improvement over our previous system [2]. The proposal uses a non-linear model based on NARMAX (Nonlinear AutoRegressive Moving Average model with eXogenous inputs), with the goal of achieving a cheap and non-intrusive gaze focalization system. It is able to achieve accuracy results on pair of most sophisticated systems over complex accidental scenarios. With this objective, the work proposed here has been validated by using the recent and challenging public database DADA2000 [3]. It collects real accidental scenarios annotated with the accident localization on the scene and the attention map generated by some users who watched the scene on their laboratory using an expensive and active desktop-mounted eye-tracker. We present a comparison with their gaze focalization system in similar conditions of the one reported by the authors, reaching on par results. From our knowledge, and without taking our previous work into account, this is the first time that a camera-based vehicle-mounted driver attention system is evaluated in this challenging database.

## 2. System Architecture

**Figure 1:**
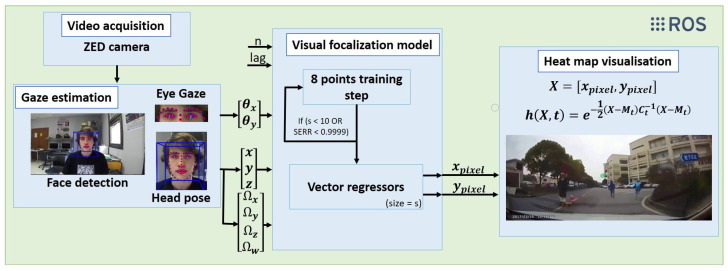
Non-linear calibration framework.

The proposed system has four sections that work together to extract the gaze focalization. These sections are: video acquisition, gaze estimation, visual focalization model and heat map visualization. The architecture is shown in Figure 1.

To communicate all the sections, a Robot Operating System (ROS) [33] is used, which is a framework that allows bastardization, communication and modularity facilities to all the sections. It offers the possibility to modularize the complete system and even allows the possibility of working on distributed systems, such as two computers.

### 2.1. Video Acquisition

The image from the user is obtained using a ZED camera from Stereolabs, which is a stereo camera. However, for this application, we will be using it as a monocular camera. It has been chosen due to its ROS compatibility and for its specs of frame rate and resolution. On the one hand, Sterolabs provides a wrapper that allows the publication of all the camera data using ROS topics. Furthermore, on the other hand, the camera is able to work at 100 fps at VGA, which is much higher than that obtained by a standard web cam. This application is not limited to the kind of camera. It could work with a standard web cam, but obtaining worse results.

An ablation study about camera parameters is quite important using the camera setup. Moreover, the relative position to the user is an interesting topic to be evaluated because of the possibilities this kind of systems provide on real environments. These studies will be carried out in the following sections.

**Figure 2:**
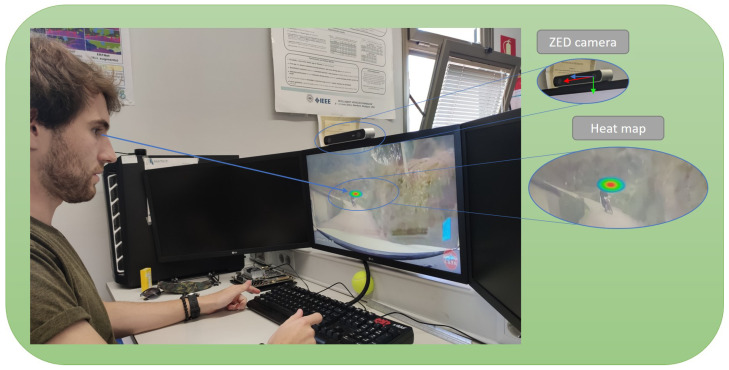
Full framework set-up. User looking at a 24${}^{\prime \prime }$ screen with the camera positioned in front of him, while DADA2000 videos are displayed on the screen. Heat map is generated in real time.

Figure 2 shows how the experiment was set-up in order to emulate the same experiment as the authors of the DADA2000 database [3] in order to have a fair comparison.

### 2.2. Gaze Estimation

The main proposal of this work is to contribute to the scientific field with a gaze focalization system based on camera. It is a non-intrusive and cheap system that does not generate rejection nor fatigue in the driver and can be used on a vehicle without disturbing the user. Therefore, desktop-mounted eye trackers and head-mounted eye trackers are refused. Gaze estimation systems based on cameras that can be used on vehicles have been proposed in the literature [6,11,12,13,14]. Face detection using video is a key task for this kind of systems. On this research field, OpenFace is one of the most popular open-source facial analysis tools because of its robustness and performance. We proposed the usage of OpenFace 2.0 toolkit [1] because it provides face detection and 68 facial landmarks. With some of these landmarks, it estimates the gaze direction, as shown in Figure 1.

The tool uses a Conditional Local Neural Field (CLNF) to detect the facial landmarks, within which are included the next eye landmarks: eyelids, iris and pupils. With the eyes detected and situated on the space, it computes the gaze vector of each eye using the eye-region. Regarding the gaze, it returns three vectors, one for each eye and a third one that is the fusion of both eyes, which will be used with other facial landmarks on this work. The authors tested performance of the tool on the challenging MPIIGaze database [34] achieving a mean absolute error of 9.1 degrees per frame. This is a satisfactory result for this benchmark and good enough for our application. The data from the 68 landmarks used on this work to achieve gaze focalization will be explained on the next sections.

### 2.3. Visual Focalization Model

Angle gaze variation is not linear, but it can be assumed to be linear when the gaze range is small. To implement a system as general as possible, we improve our previous publication based on a linear method [2], proposing the implementation of a Nonlinear AutoRegressive Moving Average model with eXogenous inputs (NARMAX model).

The NARMAX model [35] includes a wide class of nonlinear systems, which means that the modeled signal depends of:

- Past signal terms (AR);
- Past noise terms (MA);
- Other signal with a possible delay (X).

Relationships among them are nonlinear and the modeled signal is discrete, which can be defined as:

(1) $$\begin{array}{c} y\left(k\right)=F[y\left(k-1\right),y\left(k-2\right),\ldots ,y\left(k-n_{y}\right),\\ u\left(k-d\right),u\left(k-d-1\right),\ldots ,u\left(k-d-n_{u}\right),\\ e\left(k-1\right),e\left(k-2\right),\ldots ,e\left(k-n_{e}\right)]+e\left(k\right) \end{array}$$

where y(k), u(k) and e(k) are the system output, input, and noise sequences, respectively; $n_{y}$, $n_{u}$, and $n_{e}$ are the maximum lags for the system output, input and noise; F[] is some unknown nonlinear function, *d* is a time delay typically set to d=1.

NARMAX is based on five steps that answer the following questions:

1. Structure detection—“What parts are in the model?”;
2. Parameter estimation—“What are the values of the model coefficients?”;
3. Model validation—“Is the model correct and unencumbered?”;
4. Prediction—“What does the modeled signal look like in the future?”.

There are some methods that achieve these steps such as FROLS or MFROLS [36], that will be tackle hereafter. These algorithms try to find the most simple model that describes the structure of the system.

The problem of this approach is that the developer has to suggest possible types of nonlinearity because the number of nonlinearities is quite high. These can be:

- Polynomial degree;
- Term degree;
- Logarithm;
- Neuronal network;
- Superposition of the above methods.

For this reason, modeling a nonlinear system brings the possibility of unknowingly making a fatal error. If the system under test has a nonlinearity that is not assumed, the model will not be fitted the data. In NARMAX methods, the only limit is the assumption made by the modeler. Here, some nonlinear modeling systems are presented.

#### 2.3.1. OLS

This work proposes using the Orthogonal Least Squares (OLS) algorithm to establish the correspondence between the face features based on the camera and the coordinates projected by the gaze on the screen. This approach has been used on nonlinear systems because OLS will search through all possible candidate models to select the better approach.

The main idea of the OLS estimator is to introduce an auxiliary model whose elements are orthogonal to the signal that is modeled, as in the Wiener Series. Then, subsequent parts of the orthogonal model can be determined in turn, and then the parameters of the searched model can be calculated based on them. Iterative repetition of the steps of this method allows not only finding unbiased model estimators, but also showing what contribution each of them has in the final modeling result.

Considerations should begin with the assumption of the general model:

(2) $$y\left(k\right)=\sum_{i=1}^{M}\theta_{i}p_{i}\left(k\right)+e\left(k\right)$$

where y(k) is the modeled system response for k=1,2,…,N; θ are the model parameters (θ∈R) associated to the regressors $p_{i}\left(k\right)$ and e(k) is the external noise or error at the moment k=1,2,…,M.

Regressors $p_{i}\left(k\right)$ are defined as the combination of delayed signal values or delayed external signals. In the general case, the function of the delayed members of the model can take any non-linear form. For the model, it is also assumed that each regressor $p_{i}\left(k\right)$ is independent of the model parameters θ, therefore:

(3) $$\frac{\partial p_{i}}{\partial \theta_{j}}=0\;\mathrm{for}\;i=1,2,\ldots ,M\;\mathrm{and}\;j=1,2,\ldots ,M$$

The goal of the estimator is to transform the model specified in Equation (2) into an auxiliary model whose elements are orthogonal to each other. This type of model has the form:

(4) $$y\left(k\right)=\sum_{i=1}^{M}g_{i}\left(k\right)q_{i}\left(k\right)+e\left(k\right)$$

where $g_{i}$ are the parameters of the orthogonal model and $q_{i}\left(k\right)$ (i=1,2,…M) are the orthogonal components of the model. The orthogonality condition is then presented as:

(5) $$\sum_{k=1}^{M}g_{i}\left(k\right)q_{i}\left(k\right)=\left\{\begin{array}{cc} d_{i}=\sum_{k=1}^{M}q_{i}^{2}\left(k\right)\neq 0, & i=j\\ 0, & i\;!=\;j \end{array}\right.$$

The orthogonalization procedure for the model from Equation (2) can be summarized as:

(6) $$\begin{array}{c} \left\{\begin{array}{cc} q_{1}\left(k\right) & =p_{1}\left(k\right)\\ q_{2}\left(k\right) & =p_{2}\left(k\right)-a_{1,2}p_{1}\left(k\right)\\ q_{3}\left(k\right) & =p_{3}\left(k\right)-a_{1,3}p_{1}\left(k\right)-a_{2,3}p_{2}\left(k\right)\\ \; & \vdots \\ q_{m}\left(k\right) & =p_{m}\left(k\right)-\sum_{r=1}^{m-1}a_{r,m}q_{r}\left(k\right),m=2,3,\ldots ,M \end{array}\right. \end{array}$$

where parameter $a_{r,m}$ associates the components of the output model from Equation (3) with the orthogonal model according to the following form:

(7) $$a_{r,m}=\frac{\sum_{k=1}^{N}p_{m}\left(k\right)q_{r}\left(k\right)}{\sum_{k=1}^{N}q_{r}^{2}\left(k\right)},\;1\leq r\leq m-1$$

On the base:

(8) $$g_{i}=\frac{\sum_{k=1}^{N}y\left(k\right)q_{i}\left(k\right)}{\sum_{k=1}^{N}q_{i}^{2}\left(k\right)},\;i=1,2,\ldots ,M$$

Therefore:

(9) $$\begin{array}{c} \left\{\begin{array}{cc} \theta_{M} & =g_{M}\\ \theta_{M-1} & =g_{M-1}-a_{M-1,M}\theta_{M}\\ \theta_{M-2} & =g_{M-2}-a_{M-2,M-1}\theta_{M-1}-a_{M-2,M}\theta_{M}\\ \; & \vdots \\ \theta_{m} & =g_{m}-\sum_{j=m+1}^{M}a_{m,j}\theta_{j},m=M-1,M-2;\ldots ,1 \end{array}\right. \end{array}$$

This allows, based on previously established regressors, to develop a model based on orthogonal components, and to recreate the basic model from this type of model.

#### 2.3.2. ERR

The problem in carrying out OLS estimation is the criterion for selecting subsequent regressors for the orthogonal model being created. Remembering that the model also has noise *e*, the energy (or variance) of the system can be represented as:

(10) $$\frac{1}{N}y^{T}y=\frac{1}{N}\sum_{i=1}^{M}g_{i}^{2}q_{i}^{T}q_{i}+\frac{1}{N}e^{T}e$$

where *y* is the modeled signal vector, $g_{i}$ are the coefficients standing next to elements of the orthogonal model, $q_{i}$ are the vector elements of the orthogonal model, *e* is the vector of noise samples and *N* is the number of signal samples. It can be stated that:

(11) $$1=\frac{\sum_{i=1}^{M}g_{i}^{2}q_{i}^{2}q_{i}}{y^{T}y}+\frac{e^{T}e}{y^{T}y}=\sum_{i=1}^{M}ERR_{i}+ESR$$

where $ERR_{i}$ is the Error Reduction Ratio and ESR is the Error to Signal Ratio. To measure the significance of each model parameter, the Error Reduction Ratio (ERR) is used, which indicates the system improvement, in percentage (0–1). It can be accounted by including the model parameters. This capability is important for the model to get the best model, without getting a complex one. ERR allows the model to get only the best parameters in order to achieve best model performance without a high training time and without complex the model.

When the sum of subsequent values ERR tends to one means the modeling process can stop ($\sum_{i=1}^{M}ERR_{i}\to 1$). Comparison of individual ERR values for various elements of the model shows which of them are the most influenced on the signal of the designated model.

#### 2.3.3. FROLS

Using the OLS estimator and coefficient ERR describes the FROLS (Forward Regression with Orthogonal Least Squares) nonlinear modeling algorithm, a forward regression algorithm using the orthogonal sum of the least squares. It is assumed that the selection of subsequent regressors for the OLS estimator should be conditioned by the highest value ERR for a given regressor. This allows choosing the model member that reduces the modeling error to the greatest extent and to stop modeling when ESR will have a satisfactory value. In the following sections, steps in modeling according to the FROLS algorithm will be presented.

##### Step 1. Data Collection

In order to perform modeling, acquisition of the test signal waveforms is required, y(k) (X_pixel, Y_pixel), as well as external input signals, x(k) (OpenFace parameters), which affect the system under study. The signal should be collected as discrete or transformed into such a form. In addition, the recorded waveform should have as little external noise as possible and should not be subjected to filtration, which may disturb the identification process. According to the author of the method [35], if the noise presents in the signal is zero-mean white noise, it does not affect the ERR value and the identification process.

##### Step 2. Defining the Modelling Framework

Because the number of possible nonlinearities is very high, a priori assumption is required to define the search method. These requirements can be formulated answering the following questions:

- What is the maximum delay of the AR term ($n_{y}$)? (Output signal)?
- What is the maximum delay of external signals ($n_{x}$)? (Input signal)?
- What nonlinearities are predicted and what is their maximum degree (*l*)?

After the developer has provided the answer to these questions, the following parts of the model are known: y(k−1), y(k−2), …, $y(k-n_{y})$, $u_{i}\left(k-1\right)$, $u_{i}\left(k-2\right)$, …, $u_{i}\left(k-n_{x_{i}}\right)$. Where *y* is the signal under test, $u_{i}$ is one of the external input signals, $n_{y}$ is the maximum delay of the signal under test and $n_{x_{i}}$ is the maximum delay of one of the external input signals. The non-linearity that binds these members is also known, which allows the next step to be taken. In the case where the non-linearity is a polynomial, the maximum degree of non-linearity determines the maximum power occurring in this polynomial.

##### Step 3. Determination of the Regressor Vector

Knowing the modeling framework defined in the previous step, we must determine a vector containing all possible regressors for a given signal. For example, a system with one output (*y*) and one input (*u*) with $n_{y}=n_{x}=2$, where nonlinearity is modeled as a polynomial up to the second degree, l=2. Then, the regressor vector will consist of a combination of signals: y(k−1), y(k−2), u(k−1), u(k−2) according to the maximum polynomial degree. Including the constant component marked as const., regressor vector marked as *D*, will be defined as:

(12) $$\begin{array}{c} D=\{const.,y(k-1),y(k-2),u(k-1),u(k-2)\}\cup \\ \{y^{2}\left(k-1\right),y\left(k-1\right)y\left(k-2\right),y\left(k-1\right)u\left(k-1\right),y\left(k-1\right)u\left(k-2\right)\}\cup \\ \{y^{2}\left(k-2\right),y\left(k-2\right)u\left(k-1\right),y\left(k-2\right)u\left(k-2\right)\}\cup \\ \{u^{2}\left(k-1\right),u\left(k-1\right)u\left(k-2\right)\}\cup \\ \left\{u^{2}\left(k-2\right)\right\} \end{array}$$

##### Step 4. Choosing the First Element

Once the regressor vector has been specified, the database of the created model is known. Choosing the first one requires determining the value of ERR for each element of the vector $D=p_{1},p_{2},\ldots ,p_{m}$, where *m* is the number of potential regressors.

(13) $$g_{m}=\frac{y^{T}p_{m}}{p_{m}^{T}p_{m}}$$

(14) $$ERR\left[m\right]=\frac{g_{m}^{2}p_{m}^{T}p_{m}}{\sigma }$$

(15) $$l_{1}=arg\max_{1\leq m\leq M}ERR[m]$$

where $l_{1}$ corresponds to the highest regressor and it is accepted as the first element of the orthogonal model. The parameters *g* and ERR are saved.

(16) $$q_{1}=p_{l_{1}}\;;\;g_{1}=g_{l_{1}}\;;\;err\left[1\right]=ERR\left[l_{1}\right]\;;\;D_{1}=D-p_{l_{1}}$$

After saving the found regressor and its associated values, the search is restarted to find the next member of the orthogonal model.

##### Step 5. Selecting the Next Elements of the Model

The next steps are analogously performed to the first step, with the difference that the orthogonal model already has a certain number of terms, depending on the number of steps previously performed. Calculation of ERR values for potential new members of the model must be made again, in relation to the current form of the model. Therefore, the regressor index is searched (marked as $l_{s}$) about the highest ERR for the current model form, where *s*) is the current step. The regressor vector is marked as $D_{s-1}$ and does not contain the members selected in the previous steps. Then, for m=1,2,⋯,M−(s−1) and $m\neq l_{1}\neq l_{2}\neq \cdots \neq l_{s-1}$, we have:

(17) $$q_{m}=p_{m}-\sum_{r=1}^{s-1}\frac{p_{m}^{T}q_{r}}{q_{r}^{T}q_{r}}q_{r},\;p_{m}\in D_{s-1}$$

(18) $$g_{m}=\frac{y^{T}q_{m}}{q_{m}^{T}q_{m}}$$

(19) $$ERR\left[m\right]=\frac{g_{m}^{2}q_{m}^{T}q_{m}}{\sigma }$$

(20) $$l_{s}=arg\max_{1\leq m\leq M-(s+1)}ERR[m]$$

After finding $l_{s}$, which corresponds to the highest regressor ERR, the orthogonal model is extended by another member, $q_{s}$, along with the corresponding parameter $g_{s}$. The ERR value for the selected element is saved, and from the vector *D* the selected regressor is deleted. In addition, parameter values $a_{r,s}$ are determined according to the following equations:

(21) $$q_{s}=q_{l_{s}}\;;\;g_{s}=g_{l_{s}}\;;\;err\left[s\right]=ERR\left[l_{s}\right]\;;\;D_{s}=D_{s-1}-p_{l_{s}}$$

(22) $$a_{r,s}=\frac{q_{r}^{T}p_{l_{s}}}{q_{r}^{T}q_{r}},r=1,2,\cdots ,s-1$$

This step is repeated until the *ESR* value is satisfactory:

(23) $$ESR=1-\sum_{s=1}^{M_{0}}err\left[s\right]$$

where $M_{0}$ is the number of selected regressors. A certain limit is necessary for *ESR*, which will decide the end of the modeling.

#### 2.3.4. Modelling Process Analysis

An important decision when performing modeling with the NARMAX method is when to stop adding more regressors to the model. The right moment does not have a strict definition, but rather is determined by a set of factors whose superposition is a premise to take it. In the case of NARMAX, one of the basic parameters is the ERR sum for the model, called SERR:

(24) SERR=∑ERR

It is assumed that a well-fitted model has SERR higher than 0.8 [37].

#### 2.3.5. Determination of the Final Model

When $M_{0}$ regressors are selected, it is necessary to determinate their parameters. Assuming a low value of ESR, the general and ortogonalized models are equal to:

(25) $$y\left(k\right)=\sum_{i=1}^{M_{0}}\theta_{i}p_{i}\left(k\right)+e\left(k\right)=\sum_{i=1}^{M_{0}}g_{i}q_{i}\left(k\right)+e\left(k\right)$$

In the above equation, the only unknowns are $\theta_{i}$, because regressors $g_{i}$ were selected during modeling, and the elements of the orthogonal model $q_{i}\left(k\right)$ along with their parameters were appointed on an ongoing basis during the process. Therefore, it is necessary to perform the conversion from the orthogonal model to the initial model by performing an inverse process to the previous orthogonalization. Knowing the values of the elements $a_{r,s}$ for $1\leq r<s\leq M_{0}$, the following matrix is specified:

(26) $$A=\left(\begin{array}{ccccc} 1 & a_{12} & a_{13} & \cdots & a_{1M_{0}}\\ 0 & 1 & a_{21} & \cdots & a_{2M_{0}}\\ \vdots & \vdots & \ddots & \vdots & \vdots \\ 0 & 0 & \cdots & 1 & a_{M_{0}-1,M_{0}}\\ 0 & 0 & 0 & \cdots & 1 \end{array}\right)$$

which can be included in equation:

(27) Aθ=g

where $g=[g_{1},g_{2},\cdots ,g_{M_{0}}]$ and $\theta =[\theta_{1},\theta_{2},\cdots ,\theta_{M_{0}}]$. Solving this equation, model parameters are obtained, thus determining the full output model.

The output has a vector form of two coordinates (*X*_pixel, *Y*_pixel), so, two models have to be calculated in order to obtain the system output as required.

Three main parts compose the framework used on this calibration step, and how the subsystems are communicated among them in order to achieve the best result. The first one is in charge of getting data to calibrate. The second one calculates the regressors for the model. Furthermore, finally, the last one uses these regressors and sends the topic to the ROS environment.

In order to achieve this calibration method, a training method is necessary. So, at the beginning of any test, the user has to look at eight points around the screen. Points are displayed during 8 s, but in order to get a better accuracy on the model, the first two seconds are removed because in this time the eyes are in transition between fixations.

To perform this task, the eight points are displayed in red over a white image using OpenCV. These points have a 30 pixel radio and are placed on the following positions in pixels: $\left[X_{1},Y_{1}\right]=\left[200,200\right]$, $\left[X_{2},Y_{2}\right]=\left[700,200\right]$, $\left[X_{3},Y_{3}\right]=\left[1100,200\right]$, $\left[X_{4},Y_{4}\right]=\left[1700,200\right]$, $\left[X_{5},Y_{5}\right]=\left[200,700\right]$, $\left[X_{6},Y_{6}\right]=\left[700,700\right]$, $\left[X_{7},Y_{7}\right]=\left[1100,700\right]$ and $\left[X_{7},Y_{8}\right]=\left[1700,700\right]$. After training, these points will be evaluated during a testing procedure.

Data are composed of the point where the user is looking at (*X*_pixel, *Y*_pixel) and the data provided by OpenFace for the gaze and the head for this position, which is formed by:

- Gaze angle *X* ($\theta_{x}$);
- Gaze angle *Y* ($\theta_{y}$);
- Head position *X* (*x*);
- Head position *Y* (*y*);
- Head position *Z* (*z*);
- Head rotation *X* ($\Omega_{x}$);
- Head rotation *Y* ($\Omega_{y}$);
- Head rotation *Z* ($\Omega_{z}$);
- Head rotation *W* ($\Omega_{w}$).

Data are referenced to the camera and saved on a *.txt* file with the next structure: Gaze angle *X*; Gaze angle *Y*; Head position *X*; Head position *Y*; Head position *Z*; Head rotation *X*; Head rotation *Y*; Head rotation *Z*; Head rotation *W*; *X*_pixel; and *Y*_pixel. These are the parameters chosen, because gaze focalization systems take as input: eyeball orientation and head pose (orientation and position of the head) [16]. Instead of eyeball orientation, we use a gaze vector provided by OpenFace 2.0 to obtain a more accurate gaze focalization system.

**Figure 3:**
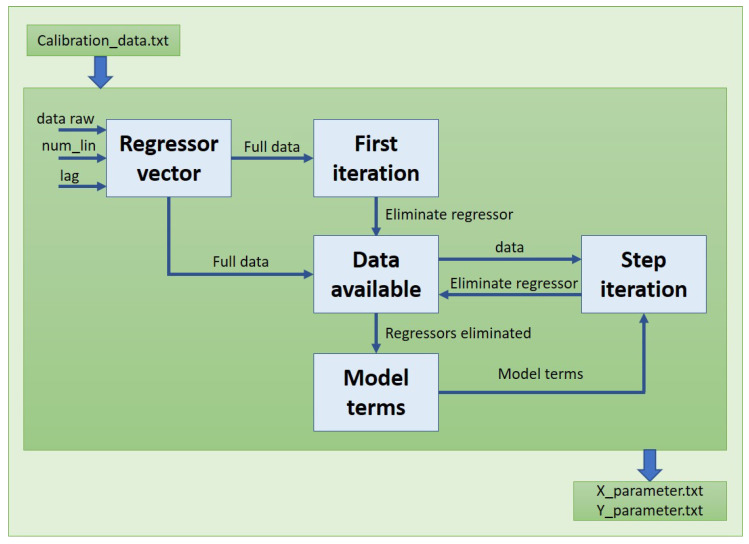
Nonlinear calibration script flow.

The file created with the data recorded for each test will be charged on the next step of the model where a training procedure is carried out to calculate the parameters, as it is defined on Figure 3, where the data feed the model and following the steps described earlier to obtain the regressors and their parameters for the two models.

NARMAX model is fit with these parameters. The model will use the parameters necessary to fit the model to the output as best as possible. After some experiments, the best option is obtained for a Sum Error Reduction Ratio (SERR) higher than 0.9999 or 10 regressors. More regressors complicate the model with a slight improvement on accuracy and over-fit the model.

### 2.4. Heat Map Visualisation

One of the most common visualization approaches to represent a spatial distribution produced by the gaze focalization on the screen and the one used on this work is the heat map. To obtain the spatial distribution over the time we have used a temporal window [t−w,t], where *t* is the actual frame and *w* is the temporal window, which has been set to 1 s as done by [38]. With this temporal window, we have 30 samples to generate our spatial distribution that will be represented as a heat map. The distribution is fitted on a Gaussian model, which represents the different areas with a color gradient. ($M_{t}=\left[m_{x}\left(t\right),m_{y}\left(t\right)\right]$) is the center of the Gaussian, $C_{t}$ is the covariance matrix and h(X,t) is the intensity of the heat map for the pixel (X=[x,y]). For this work, we have drawn ellipses following the Gaussian model with two times the standard distribution, using the next equation:

(28) $$h\left(X,t\right)=e^{-\frac{1}{2}{\left(X-M_{t}\right)}^{T}C_{t}^{-1}\left(X-M_{t}\right)}$$

## 3. Experimental Results

In this section, we explain and expose the results obtained for our architecture proposal using the non-linear calibration method based on NARMAX. The evaluation is divided into two different subsections. Firstly, a precision experiment is done to test the accuracy of the system. Furthermore, secondly, a comparison is conducted with other state-of-the-art systems in the challenging database DADA2000 as we carried out in [2].

### 3.1. Visual Focalization Model Evaluation

To evaluate the precision of our driver focalization system, we have applied the same testing procedure as done previously with the linear calibration. After the calibration and training step, the user has to focalize their gaze on eight points during eight seconds per point, that are displayed on different locations over the screen. The first second of each point has been despised because the gaze is not stable when transitioning to the new position to be glanced. This test has been done in the ablation study to test the best camera parameters, the best camera position and the loss of accuracy that the use of glasses may cause to the system. After that, it has been carried out with some users to evaluate the system. To evaluate the accuracy of the system, we have used the Root Mean Square Error (RMSE) between the gaze estimation on the screen and the localization of the points. Accuracy is shown in total and regarding the (*X*, *Y*) axes, and is evaluated in percentage, pixels and millimeters.

### 3.2. Camera Parameters

The ablation study starts by analyzing the camera parameters that best suit the system. We have evaluated different resolutions and camera frame rates and tested the precision of the system for all these parameters. The camera used ZED from Sterolabs, which allows four different resolutions with different frame rates, as shown in Table 1. On the performance column, we show the ability of OpenFace to process frames. The test was repeated by the same user for 5 times in order to achieve a better conclusion about the best camera parameters to be used for the next sections.

This experiment shows that the *X* axis gets the best accuracy using 2k resolution at 15 frames per second. Instead, the *Y* axis gets the best accuracy using HD1080 at 30 frames per second. Taking into account the two axes, the best accuracy is obtained for HD1080, which is different to the linear calibration results obtained in previous works, where best camera parameters were 720p at 60 frames per second. In conclusion, HD1080 at 30 frames per second will be the camera parameters used for the next tests performed with the NARMAX calibration.

Results show a quite improvement from the linear calibration method, which obtained an error of 60.5 pixels, 3.5 times higher than the obtained with this nonlinear method.

### 3.3. Camera Position

Camera position is vital for the system to work, as it gives the opportunity to place the camera on different locations in the vehicle. We are going to repeat the study as in the linear calibration where the camera was placed in front of the user at different heights (10, 48 and 78 cm) over the table where the screen was located.

Table 2 shows the results obtained for this test. The results are displayed in the same conditions as done in our previous work. The best accuracy is obtained when the camera is just in front of the user at 48 cm above the table. Nevertheless, this localization is not possible in a real car as it will hide the road scene. Placing the camera on the top localization is not allowed because most of the landmarks of the face are occluded and OpenFace 2.0 does not work properly, as happened with the linear calibration. This problem cannot be solved with this new calibration method because OpenFace loses some landmarks and the input model is already wrong. Therefore, the bottom position has been chosen to place the camera for future real applications on a vehicle, as was done with linear calibration.

Even though the bottom position is the most suitable for a real application on the vehicle, the middle position is used for the next tests, due to the fact that this middle position is the best one for in-lab application. Results show an improvement of more than 6 times for the bottom position and near 3.5 for the middle position.

### 3.4. Glasses

Once the ablation study has been carried out, it is time to test the performance when the user wears glasses as some drivers wear glasses while driving in a mandatory way, whereas others prefer to drive with sunglasses. The results previously obtained with the linear calibration method showed that it cannot work with a user wearing sunglasses, which limits the use of the previously proposed system.

NARMAX calibration method shows that performance is even better using glasses than without them. This could be because glasses are rigid elements where OpenFace is able to find robust visual features. Even so, differences between them are less than a pixel, which are no representatives. Both tests were done using uniform lighting conditions, which will differ in a real experiment where the sun may cause sun-glasses on the glasses. Results are depicted on Table 3. With this calibration method, it is possible to track the gaze even with sunglasses This is because this process takes into account face parameters, in addition to the gaze ones, which in this case are occluded. We have obtained an improvement over the linear calibration between 3 and 4 times better.

### 3.5. Precision Test

**Figure 4:**
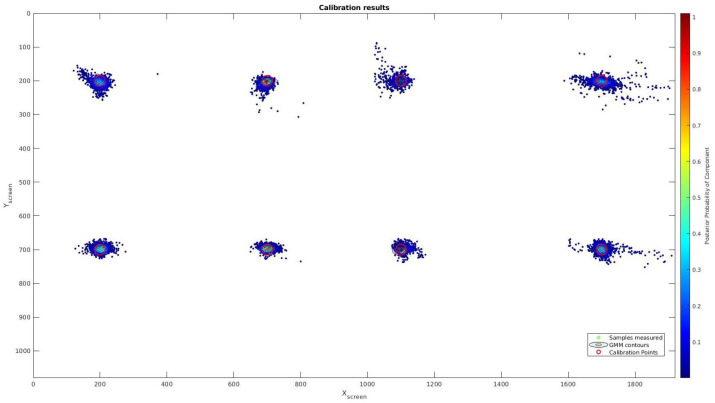
Calibration results using NARMAX calibration method. Eight points are displayed on the screen (Red circle). The results from our output creates 8 Gaussian around the testing points.

After the ablation study and the opportunity to work with any kind of user indistinctly if they wear glasses or not, we have performed an evaluation with some users to get statistical results. The test was done by 10 users who calibrated the system following the procedure explained early to perform the test and obtain a comparison with the method presented in [2] in terms of precision. Results for the whole users can be seen in Figure 4.

**Figure 5:**
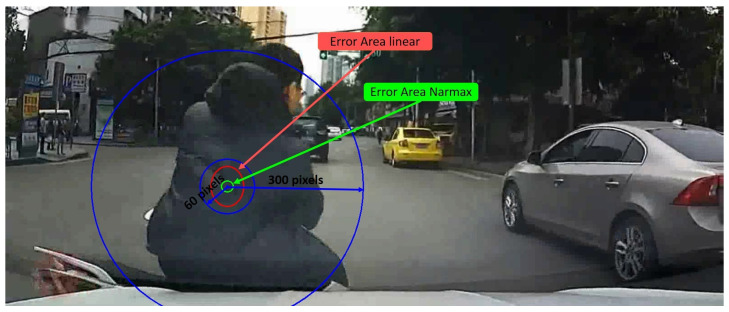
DADA2000 frame. Crash object circle for 60 and 300 pixels (blue). Error linear calibration area (red). Error NARMAX calibration area (green).

Our method achieves about 1.02% error on the X axis and 1.04% error on Y axis, which is an error 3 times less than the obtained by the linear calibration as can be seen in Table 4 Unlike linear calibration method, this system provides a similar accuracy in both axes which is an important improvement because it allows having circular focalization map instead a ellipsoids one, which is the correct approximation for a focalization error. Figure 5 shows the error overprinted on an image with a crash object in order to compare scales for the both tested systems. Furthermore, the linear calibration error is displayed in order to check the difference between both methods. This new model offers a better tool to obtain the focalization map generated by a driver.

### 3.6. DADA2000 Evaluation

The presented system based on NARMAX model shows potential to be a satisfactory focalization method in order to be used for driving application. However, it has to be firstly tested on a challenging video benchmarking (DADA2000), which is composed by accidental videos including driver attention and annotated accident localization. This allows evaluating the performance of our proposal to estimate accidents in video sequences as done with the linear calibration.

We are going to compare the results obtained by our system with those found by the authors of the database (DADA2000) using an expensive and active desktop-mounted eye-tracker. Some users watched the same videos in similar conditions as done with the linear calibration.

**Figure 6:**
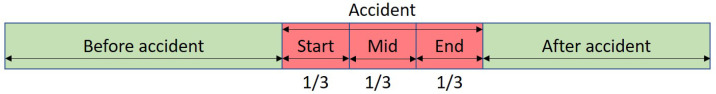
Temporal partition of the video. Dividing the accident part in three pieces of same length to evaluate the results by sections.

This database provides with three annotated information pieces. Firstly, it offers the crash-object position (accident localization in pixel per frame). Secondly, it contributes with the focalization map captured with a desktop-mounted eye-tracker by some users. Furthermore, lastly, the temporal window that involves the accident is exposed. The provided videos are partitioned in three main clips: before the accident, during the accident and after the accident, as it is depicted in Figure 6. The accident clip is also divided into three sub-clips (Start, Mid and End) for a better analysis.

#### 3.6.1. Crash Object Detection in DADA2000 Benchmark

Crash object detection has been tested with four users who were invited to watch the videos, in the same way as done using the linear calibration. Between videos, users made a pause of 3 s to accommodate the gaze for the next video. The database was resized from 1584 × 660 to 1920 × 1080 to match it with the screen resolution used in the experiment by the original authors, in order to wide the working field of view.

The clips were played at 30 fps to compare our results with the obtained by the authors of DADA2000 [3] in the same terms. We recorded attention map without temporal aggregation, obtaining one measure per frame, because with this calibration method we work at 30 frames per second, which gives one focalization measure per frame.

This test is not focused on what kind of accidents the users focalize better. This test is done in order to validate the possibility of using this method to acquire driver focalization. Because the main proposal of this work is to present a gaze focalization system, the goal of this test is to localize driver attention on a screen. For this purpose, we have used the same evaluations done by the original authors of the database. They measured the distance between the crash object center, which is manually annotated, and the attention point obtained for each frame. After that, they used some thresholds (Th) in pixel to establish if the detection is below that Th or not. With these premises we obtain two indicators (frame ratio and success rate).

Results are compared with the obtained by DADA2000 authors using a Senso Motoric Instrument (SMI) RED250 desktop-mounted infrared eye tracker, for different detection area sizes as is shown on Table 5. Our success rate is higher than the obtained by the DADA200 authors for all the partitions except for start clips at threshold between 100 and 300. However, our numbers are obtained for four users per video instead of the five they argue to use, even though they also argue every video was watched by at least two people, indicating that not all of them were watched by the five users.

For a 300 pixels threshold, we have obtained more than a 90% success rate for mid and end sections of the accident. This test shows worse results on the start section, but close to the results claimed by the authors of the database. Mid and end sections show better results than those claimed by the authors.

Results show that early accident detection is worse than for the middle and end partitions because users take some time to react and focus their gaze in the accident area. In the last part of the accident, users using NARMAX method focus their eyes in the accident area longer that with the linear method.

Table 6 shows the Frame ratio, which compared the distance between the focalization and the accident frame by frame.

**Figure 7:**
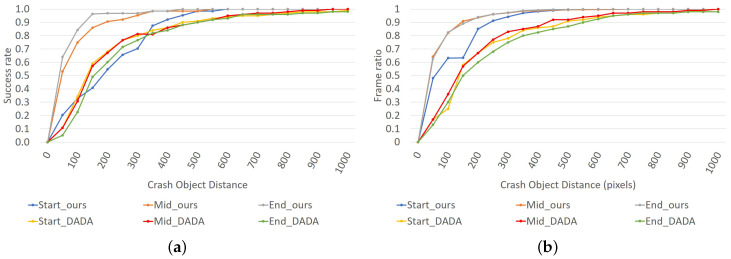
Results obtained on the testing using NARMAX calibration. (**a**) Success rate obtained by the system proposed using NARMAX calibration, (**b**) frame ratio obtained by the system proposed using NARMAX calibration.

The success rate and frame ratio curves are shown on Figure 7, this figure shows the curves obtained for the different Th (60 to 1000 pixels) and divided by the three sub-clips in which the accident is divided, as done by the original author in [3]. Our results outperform the obtained for our baseline for all the analyzed sub-sections except the success rate start section, which has a lower curve in some sections of the curve (160–400 pixels).

#### 3.6.2. Heat Focalization Map on DADA2000

Heat focalization map is the visualization tool used for this method, as done with the linear calibration for analyzing driver gaze focalization areas. For this purpose, it is necessary to establish a temporal window which has been set to 1 s, as done in the DR(eye)VE project. This window represented 60 measures using the linear calibration, but as this method works better with 30 frames per second, in this case has been set to 30 measures.

The way this experiment has been done is showed on Figure 2, where the user under test is looking at the accident while the system is evaluating their gaze focalization over the accident displayed and recording results in real time. The attention map generated by the samples is shown. As we can see, our focalization map is included in the baseline map, showing the usability of our system but using a cheaper and non-intrusive sensor than the authors of the database.

**Figure 8:**
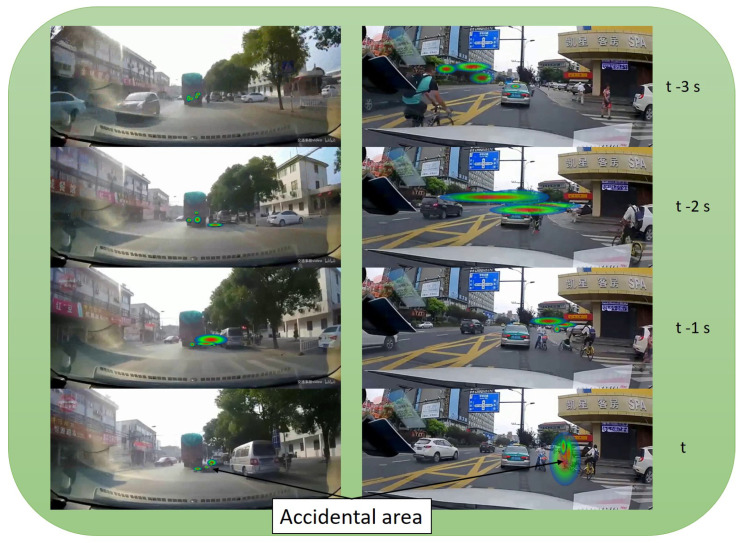
Attention map with temporal aggregation for the frame t, t − 1 s, t − 2 s and t − 3 s using NARMAX calibration method.

Figure 8 shows how temporal aggregation on heat attention map can help to predict where the accident will happen. Displayed accidents involved other vehicles were the ego-vehicle is an observer of the accident. This figure shows the heat map created by four users. The figure shows two accident situations with their corresponding focalization maps in the image where the accident takes place as a heat map. Images are displayed with a temporal difference of 1 s. As we can see, the heat map is able to correctly predict all the crash object position. Being able to generate focalization maps is the key of this work in order to get where drivers look at when they are driving as a way to predict their driving behaviors in the near future

## 4. Conclusions and Future Works

This paper presents a new low-cost and non-intrusive method to obtain visual focalization maps using NARMAX method as an improvement over our work proposed on [2].

Our system is composed of a passive camera that can be mounted on every vehicle. This system is satisfactory enough to be an alternative to active desktop-mounted eye trackers (our baseline) or head-mounted eye-tracker, which are intrusive, costly and difficult to be mounted in a real vehicle. We have validated our system in a challenging public database DADA2000 [3] showing on par results with our baseline (desktop-mounted eye trackers) in similar conditions. It has been exposed that the proposed system could be a competitive technique to monitor driver focalization over other state-of-the-art systems. It is able to be used on real applications such as the take over request maneuver or driving environment awareness in intelligent vehicles. It contributes with a security complement to the vehicle in order to make a safer driving for all the users.

As future works, we plan to use the presented system for a take over request experiment and to record an attention database with the attention map generated by this system instead of the active desktop-mounted eye tracking used in the current state of the art. Moreover, this is a excellent approach to be tested on our current simulator based on CARLA [39].

## Figures and Tables

**Table 1:** Testing accuracy vs. camera parameters on a 1920 × 1080 screen using NARMAX calibration method. Test done by one person looking at eight points on a screen. Test was done 5 times.

Method	Resolution	Frame	Acc_x			Acc_y			Acc_Total			Performance
		Rate	%	pix	mm	%	pix	mm	%	pix	mm	(Hz)
NARMAX	HD2K	15	**0.7**	12.5	3.9	1.3	14.5	4.5	1.1	20.3	6.3	14.8
NARMAX	HD1080	30	0.7	12.6	3.9	**1.1**	11.8	3.7	**0.9**	17.3	5.4	29.47
NARMAX	HD720	60	1.6	29.9	9.3	0.9	10.2	3.2	1.3	24.7	7.7	53.447
NARMAX	VGA	100	0.9	18.2	5.7	1.1	12.4	3.9	1.1	20.2	6.3	76.16
Linear	HD720	60	1.9	36.1	11.3	4.0	43.7	13.6	3.2	60.5	18.9	50.74

**Table 2:** Testing accuracy vs. camera position on a 1920 × 1080 screen. Test done by one person looking at eight points on a screen. Test was done 5 times.

Method	Position	Acc_x			Acc_y			Acc_Total		
		%	pix	mm	%	pix	mm	%	pix	mm
NARMAX	Top	-	-	-	-	-	-	-	-	-
NARMAX	Mid	**0.7**	12.6	3.9	**1.1**	11.8	3.7	**0.9**	17.3	5.4
NARMAX	Bot	1.0	18.6	5.8	1.5	16.1	5.0	1.3	24.1	7.5
Linear	Mid	1.9	36.1	11.3	4.0	43.7	13.6	3.2	60.5	18.9
Linear	Bot	2.5	48.0	15.0	10.6	114.5	35.8	7.7	147.9	46.2

**Table 3:** Testing accuracy vs. glasses on a 1920 × 1080 screen. Test done by one person looking at eight points on a screen. Test was done 5 times using NARMAX calibration.

Method	Eyes-Glasses	Acc_x			Acc_y			Acc_Total		
		%	pix	mm	%	pix	mm	%	pix	mm
NARMAX	Free	0.7	12.6	3.9	1.1	11.8	3.7	0.9	17.3	5.4
NARMAX	Glasses	**0.6**	11.6	3.6	**1.0**	11.3	3.5	**0.9**	16.4	5.1
NARMAX	Sunglasses	0.9	17.5	5.5	1.3	13.6	4.2	1.1	21.1	6.6
Linear	Glasses	2.5	48.7	15.2	4.6	50.0	15.6	3.7	71.7	22.4

**Table 4:** Testing accuracy on a 1920 × 1080 screen. Test done by 10 users looking at eight points on a screen using the NARMAX calibration method.

Method	Users	Acc_x			Acc_y			Acc_Total		
		%	pix	mm	%	pix	mm	%	pix	mm
NARMAX	10 people	**1.02**	19.5	10.4	**1.04**	11.2	6.0	**1.03**	19.7	6.2
Linear	25 people	1.9	36.1	11.3	4.0	43.7	13.6	3.2	60.5	18.9

**Table 5:** Success rate: percentage of number of clips that frame ratio for the entire clip is more than half of the frames of the entire accident scene.

System	Ours (Based on OpenFace Using NARMAX Calibration)			DADA2000		
Th (pixels)	Start	Mid	End	Start	Mid	End
60	20.3%	53.1%	64.1%	10.8%	10.7%	5.1%
100	32.8%	75.0%	84.4%	34.4%	30.8%	22.6%
160	40.7%	85.9%	96.3%	59.0%	57.2%	49.1%
200	54.7%	90.6%	96.9%	68.0%	67.2%	60.1%
260	65.6%	92.2%	96.9%	76.6%	76.6%	71.5%
300	70.3%	95.3%	96.9%	80.0%	81.3%	76.6%
360	87.5%	98.4%	98.4%	84.0%	81.0%	82.0%
400	92.2%	98.4%	98.4%	86.0%	86.0%	84.0%
460	95.3%	98.4%	100%	90.0%	88.0%	88.0%

**Table 6:** Frame ratio: percentage of frames in each clip section where the attention point is inside the correct detection area. This is measured frame by frame because the crash object change with it.

System	Ours (Based on OpenFace Using NARMAX Calibration)			DADA2000		
Th (pixels)	Start	Mid	End	Start	Mid	End
60	48.0%	64.3%	62.9%	17.0%	17.0%	13.0%
100	63.2%	82.0%	82.5%	25.0%	36.0%	30.0%
160	63.4%	90.9%	89.1%	58.0%	57.0%	50.0%
200	85.2%	93.6%	94.0%	67.0%	67.0%	60.0%
260	91.3%	96.2%	96.1%	75.0%	77.0%	68.0%
300	94.2%	97.2%	97.3%	78.0%	83.0%	75.0%
360	97.1%	98.4%	98.8%	84.0%	85.0%	80.0%
400	98.2%	98.8%	99.1%	86.0%	87.0%	82.5%
460	99.0%	99.4%	99.6%	87.0%	92.0%	85.0%

## Data Availability

Not applicable.
